# Appraisal of Sexual Education Curriculum in Secondary Schools With Inclusion of Practical Implications and Evaluation, Based on Rural-Urban Residence

**DOI:** 10.7759/cureus.80845

**Published:** 2025-03-19

**Authors:** Alexandria L Betit, Christina Kennedy

**Affiliations:** 1 Physiology, Alabama College of Osteopathic Medicine, Dothan, USA

**Keywords:** adolescents, comprehensive sexual education, contraception, curriculum, high school, middle school, rural, sexual health, urban

## Abstract

Introduction

The primary objective of this study was to review the comprehensibility of sexual education content and the age at which this curriculum was received by participants within middle and high school through a descriptive study on osteopathic medical students. The secondary objective involves a comparison of sexual education content within middle and high schools based on rural-urban residence.

Methods

An IRB-approved survey was created utilizing Qualtrics software and was administered to school-issued email accounts of Alabama osteopathic medical students within the classes of 2023 through 2026. Responses were recorded over a six-month time frame with a collection of 140 responses. Statistical analyses were completed utilizing Prism 10 software. Fisher exact testing was performed given the small sample size of survey respondents.

Results

Survey respondents attended middle and high schools throughout the United States. Most participants attended an urban middle school (87.14%; N=122) and an urban high school (90.00%; N=126). Most participants received sexual education and information about contraception within the ninth grade (44.29%; N=62) and during middle school at ages 10 to 14 (78.57%; N=110), respectively. A little over half of students (57.45%; N=80) indicated that they thought the sexual education provided to them within middle and/or high school was presented to them at an early enough age. The most common contraceptives that were discussed involved information about the usage, safety, and/or effectiveness of condoms (40.00%; N=56 in middle school and 55.00%; N=77 in high school; p=0.0165) and oral contraceptives (OCPs) (17.86%; N=25 in middle school and 32.86%; N=46 in high school; p=0.0058). All contraceptive methods were taught more frequently in high school as opposed to middle school. A lower percentage of students received instruction regarding male and female anatomy and physiology within middle school in rural vs urban areas (33.33%; N=6 in rural areas and 62.30%; N=76 in urban areas; p=0.0378). Students received most sexual education information from online websites (17.86%), during college (17.14%), or during high school (16.43%). Top additional comments mentioned that sexual education programs should be more comprehensive (50.00%) and need continuity (29.17%).

Conclusion

As supported by prior literature, despite participants’ high education level, we can assume that comprehensive sexual education reform is necessary and may be initiated within middle school with continuation into high school. Efforts should be made to incorporate online resources into future formal secondary sexual education programs. Special attention should be made to ensure greater inclusivity along with language modifications to create safe spaces for adolescents to discuss sexual health. There was no statistically significant difference in contraception education between rural and urban areas. Consequently, there can be no conclusions made suggesting that contraceptive education is lacking for those individuals living in rural vs urban regions. Future studies should aim to expand the number of survey participants across the United States within a setting other than medical school, such as within large undergraduate institutions. These institutions can gather individuals from diverse rural-urban residences and include students who are closer in time to their formal secondary school sexual education instruction.

## Introduction

Sexual education has covered an array of topics with goals focused on sexually transmitted disease (STD) and teenage pregnancy prevention. The Centers for Disease Control and Prevention (CDC) Health Education Curriculum Analysis Tool (HECAT) recommended including education regarding contraception in the eighth grade [1]. However historically, some formal sexual education curricula teach abstinence-only or abstinence as a method of teenage pregnancy prevention with little or no inclusion of contraception information [2]. Additionally, as of October 2020 only 30 states and the District of Columbia require that public schools teach sexual education curricula [3]. Of those 30 states, only four (Hawaii, Illinois, Maine, and Missouri) explicitly require the inclusion of contraceptive methods when sexual education courses are taught [3]. The primary objective of this study was to review the comprehensibility of sexual education content as well as the age in which this curriculum was received by participants within middle and high school through a descriptive study on osteopathic medical students. There have been no known descriptive studies that evaluate sexual education initiation and content from the perspective of medical students. Through completion of this study, one goal is to create improved knowledge regarding this health topic ultimately resulting in expanded access to sexual and contraception education resources for youth within middle and high school.

It has been reported that there were higher teen birth rates within rural areas when compared to urban areas [4]. In one study, adolescents 15 to 19 years old who lived in rural areas were more likely to not receive any sexual education whereas those who lived in urban areas were more likely to receive comprehensive sexual education which included contraception [5]. Other studies have reported differences between the age of first vaginal sex with a male partner in rural versus urban regions [4,6]. The results of these studies indicated that rural women had their first sexual encounter earlier when compared to urban women and ultimately concluded that these differences may in part be due to differing sexual and contraceptive education services provided in these rural and urban regions [6]. Although these studies indicate differences in sexual behaviors between women in urban and rural areas, there are no known studies that focus on differences in contraception education between urban and rural areas. Therefore, a secondary objective of this project was to determine if there were differences that existed in contraception education within rural and urban areas. The information from this descriptive study may further emphasize the need for more effective contraceptive education information directed at schools specifically within rural or urban regions.

## Materials and methods

Data and sample

We created an original survey utilizing Qualtrics (Provo, UT) software. This electronic survey was administered to the school-issued email accounts of Alabama osteopathic medical students and responses were recorded over a six-month time frame and were stored within Qualtrics. All included participants were within the classes of 2023, 2024, 2025, and 2026 (approximately 800 medical students), over the age of 18, and attended middle and high school within the United States. Out of the approximately 800 osteopathic medical students to which the survey was dispersed, we received 140 responses. A total of 140 participants completed the survey questionnaire and were accounted for in the sample. There were no incentives offered for research participation. The Institutional Review Board at the Alabama College of Osteopathic Medicine approved (Approval No.: HS220722-E, dated July 25, 2022) this study on the basis that it met Exempt Category 2.

Instrument

The original survey consisted of 12 main questions with additional follow-up questions when appropriate and was stored and accessed within Qualtrics (see Appendices). We first asked demographic questions including year in osteopathic medical school, sex assigned at birth, and current age range. Next, questions assessed the state and rural-urban residence in which participants attended both middle and high school and whether these schools were public or private. Participants were asked at which grade level(s) sexual education and at what age contraception education was presented to them within their middle and/or high school curriculum. They were then given the opportunity to delineate through a selection option what specific sexual education information was taught within their middle and high school’s sexual education curriculum. Selection items included the prevention of sexually transmitted diseases (STDs), male and female anatomy and physiology, abstinence as a method to prevent pregnancy, abstinence was stressed as the most appropriate way to prevent pregnancy, how to choose which contraceptive method was right for them, and information about the usage, safety, and/or effectiveness of multiple different types of contraception including condoms, female condoms, the contraceptive diaphragm, spermicide, the vaginal sponge, the contraceptive injection (the Depo-Provera shot), hormonal and copper intrauterine devices (IUDs), subdermal implants, oral contraceptives (OCPs)/oral birth control, emergency contraception (the morning after pill), transdermal patches, vaginal rings, and permanent methods of contraception such as vasectomy and tubal ligation. The survey also questioned where participants received most information about topics related to sexual education including contraception education with options including middle school, high school, college, through a friend, social media, online websites, doctor’s office, and others where participants were given the opportunity for free text response, and none of the above (participant did not learn about sexual education). Participants were then asked if they believed the sexual education at their middle and/or high school was presented to them at an early enough age. If respondents selected ‘no’, they were asked at which age they think they should have been exposed to sexual education/contraception education with options to choose from ages nine through 19. Lastly, respondents were allowed to leave any additional comments pertaining to the research topic or survey. Participants were not required to answer every question.

Analysis

Analyses were completed utilizing both Qualtrics and Prism 10 (GraphPad Software, San Diego, CA) software. Qualtrics was utilized to obtain the raw number of students selecting each answer to survey questions for subsequent analyses via Prism 10 software and to perform manual percentage analyses for sexual education content. All manual percentage analyses were calculated based on 140 respondents given that this was the total number of survey respondents. Survey responses were filtered via Qualtrics based on self-reported rural-urban residence in middle school and high school to determine the number of students comprising each category. Participants were given the 2010 Census urban-rural classification where a rural area was defined as an area with a population of less than 2,500 people and an urban area was defined as including both urban clusters and urbanized areas with a population of greater than 2,500 [7]. Prism 10 software was utilized to perform all statistical analyses. Statistical analysis was performed to compare sexual education content in middle and high school as well as to compare rural middle school sexual education content to urban middle school sexual education content and rural high school sexual education content to urban high school sexual education content. Fisher exact testing instead of Chi-square testing was performed given the small sample size of survey respondents.

## Results

Across the United States, only 30 states mandate that schools have sexual education programs, 39 states require abstinence be taught, and only 20 require inclusion of contraception [2]. Regionally, five out of nine states within the Northeast, 11 out of 16 states in the South, four out of 12 states in the Midwest, and nine out of 13 states in the West mandate sexual education programs [8]. Although these mandates exist, there are no known laws that directly prohibit schools in states without sexual education requirements from implementing a general sexual education curriculum [8].

**Figure 1:**
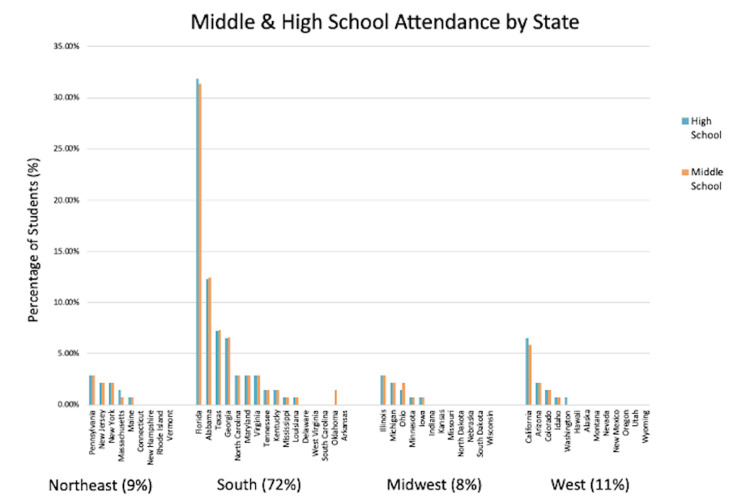
Regional middle and high school attendance of survey respondents. Most respondents attended middle and high school within the South (72%), predominantly within Florida and Alabama. Fewer participants were educated within the Northeast (9%), Midwest (8%) and West (11%). Percentages accompanying each region are depicted as averages of middle and high school attendance rounded to the nearest whole percent.

We received 140 completed survey responses via Qualtrics where participants reported attending middle and high school throughout all four regions of the U.S. within both urban and rural areas (Figure 1). Of the 140 survey respondents, 29 (20.71%) and 111 (79.29%) received their middle and high school education from a private or public institution, respectively. Among the participants, 9%, 72%, 8%, and 11% reported they attended a middle or high school within the Northeast, South, Midwest, or West, respectively (Figure 1). When assessing rural-urban residence, most participants (87.14%; N=122) attended an urban middle school while fewer (12.86%; N=18) attended a rural middle school. Within high school, 90% (N=126) of students attended within an urban area and 10% (N=14) in a rural area.

**Figure 2:**
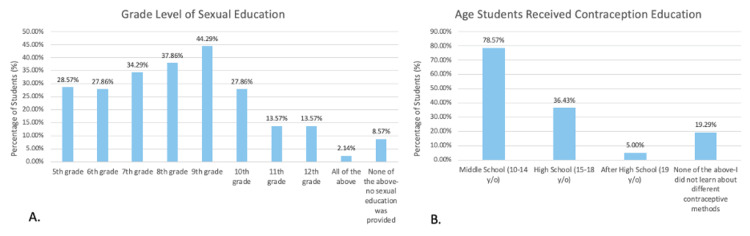
(A-B) Grade and age in which surveyed students received instruction regarding sexual education and contraception methods. Most surveyed students received information regarding sexual education in seventh, eighth, and ninth grade (A), while most received education pertaining to various contraception methods within middle school (10-14 years old) corresponding to grades five through nine (B).

Survey respondents reported the grade levels in which they received sexual education as well as the ages in which they received contraception education (Figure 2A-2B). Most participants received a sexual education curriculum within the ninth grade (44.29%; N=62), less than 3% (2.14%; N=3) of participants received sexual education throughout the entirety of middle and high school, and more than 8% (8.57%; N=12) did not receive sexual education at all during middle or high school (Figure 2A). Surveyed students did receive information exclusively pertaining to contraception education (Figure 2B). Most surveyed students received information about contraception during middle school at ages 10 to 14 (78.57%; N=110), while fewer students learned about contraception during high school at ages 15 to 18 (36.43%; N=51) and after high school at age 19 (5.00%; N=7) (Figure 2B). Over 19% (19.29%; N=27) of student participants did not receive any contraceptive education at any point within or after middle school (Figure 2B).

**Figure 3:**
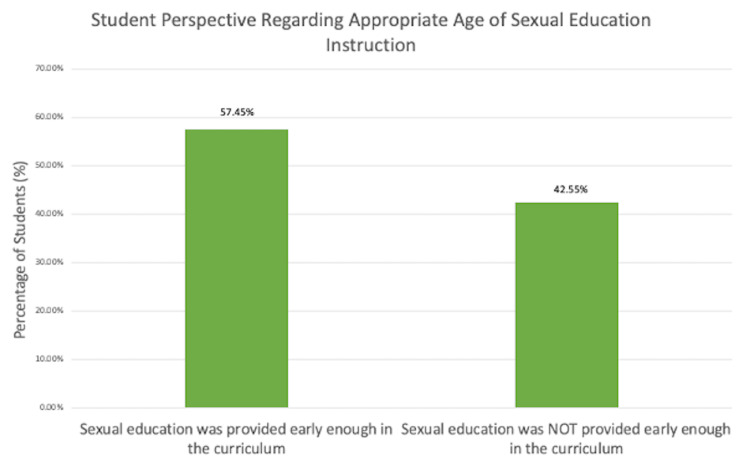
Student’s evaluation of the age of sexual education instruction. Among the students, 57.45% stated sexual education was provided to them early enough in the curriculum and 42.55% stated that sexual education was provided too late in the curriculum. Of a total of 59 respondents who stated they believed their sexual education was provided too late, 44.07% suggested early exposure around age 12.

A little over half of students (57.45%; N=80) who participated in this survey indicated that they thought the sexual education provided to them within middle and/or high school was presented to them at an early enough age (Figure 3). The 44.07% (N=59) of students who stated their sexual education was not provided early enough in the curriculum believed their curriculum exposure should have begun at age 12 (Figure 3).

Surveyed students received differing depths of sexual education content within middle and high school (Table 1). Although not statistically significant, most students were taught general sexual education content such as abstinence as a method to prevent pregnancy, male and female anatomy and physiology, and abstinence as the most appropriate way to prevent pregnancy (p>0.05) (Table 1). More students were taught about STD prevention in high school than in middle school (p<0.001) (Table 1). Students received less frequent instruction on different contraceptive methods (p<0.05) (Table 1). However, when taught, more students received this instruction in high school than in middle school (see p-values in Table 1). Predominantly in high school, the most common contraceptives that were discussed involved information about the usage, safety, and/or effectiveness of condoms (p=0.0165) and OCPs (p=0.0058) (Table 1). A smaller percentage of students received information regarding how to choose which contraception method would be right for them in middle school compared to high school (p=0.0344) (Table 1).

**Table 1:** Sexual education content provided to students through middle and high school curriculum. This table illustrates the sexual education content included in surveyed student’s middle and high school’s curricula. The number one subject taught within surveyed students' middle school sexual education curriculums was abstinence as a method to prevent pregnancy (60.00%). The next most common subjects were male and female physiology (58.57%) and abstinence being stressed as the most appropriate way to prevent pregnancy (50.71%). A smaller percentage of students reported receiving information regarding the usage, safety and/or effectiveness of contraceptive methods in middle school with the most common discussion including condoms (40.00%) and oral contraceptives (17.86%). The most common subject taught within surveyed students’ high school sexual education curriculum was abstinence as a method to prevent pregnancy (66.43%) and STD prevention (66.43%). The next most common subject was male and female anatomy and physiology (62.14%). A smaller percentage of students reported receiving information regarding the usage, safety and/or effectiveness of contraceptive methods with the most common discussion including condoms (55.00%) and oral contraceptives (32.86%). Respectively, 3.57% and 10.71% of surveyed students’ middle and high school curriculums included information regarding how to choose which contraceptive method would best suit their needs.

Sexual education content	Percentage of students (%)		
	Middle school	High school	p-value
Abstinence as a method to prevent pregnancy	60.00 (N=84)	66.43 (N=93)	0.3215
Male and female anatomy and physiology	58.57 (N=82)	62.14 (N=87)	0.6252
Abstinence was stressed as the most appropriate way to prevent pregnancy	50.71 (N=71)	49.29 (N=69)	0.9049
Prevention of sexually transmitted diseases (STDs)	40.71 (N=57)	66.43 (N=93)	<0.0001
Information about the usage, safety and/or effectiveness of condoms	40.00 (N=56)	55.00 (N=77)	0.0165
Information about the usage, safety and/or effectiveness of oral contraceptives (OCPs) (oral birth control)	17.86 (N=25)	32.86 (N=46)	0.0058
Information about the usage, safety and/or effectiveness of female condoms	10.71 (N=15)	20.71 (N=29)	0.0319
Information about the usage, safety and/or effectiveness of hormonal intrauterine devices (IUDs)	9.29 (N=13)	20.00 (N=28)	0.0172
Information about the usage, safety and/or effectiveness of the contraceptive diaphragm	8.57 (N=12)	17.86 (N=25)	0.0331
Information about the usage, safety and/or effectiveness of spermicide	8.57 (N=12)	17.86 (N=25)	0.0331
Information about the usage, safety and/or effectiveness of emergency contraception (the morning after pill)	8.57 (N=12)	21.43 (N=30)	0.0040
Information about the usage, safety and/or effectiveness of copper intrauterine devices (IUDs)	5.71 (N=8)	17.14 (N=24)	0.0042
Information about the usage, safety and/or effectiveness of contraceptive injections (the shot)	5.00 (N=7)	16.43 (N=23)	0.0032
Information about permanent methods of contraception including vasectomies and tubal ligation	4.29 (N=6)	15.00 (N=21)	0.0039
Information about the usage, safety and/or effectiveness of vaginal rings	3.57 (N=5)	11.43 (N=16)	0.0213
How to choose which contraceptive method was right for you	3.57 (N=5)	10.71 (N=15)	0.0344
Information about the usage, safety and/or effectiveness of the vaginal sponge	2.86 (N=4)	11.43 (N=16)	0.0091
Information about the usage, safety and/or effectiveness of subdermal implants	2.86 (N=4)	9.29 (N=13)	0.0424
Information about the usage, safety and/or effectiveness of transdermal patches	1.43 (N=2)	7.14 (N=10)	0.0346

Based on the 2010 Census urban-rural classification, a rural area was defined as an area with a population of less than 2,500 people, and an urban area was defined as including both urban clusters and urbanized areas with a population of greater than 2,500 [7]. Of the 140 survey respondents, 18 and 122 reported they attended a rural or urban middle school, respectively; 14 and 126 respondents reported they attended a rural or urban high school, respectively.

Information regarding sexual education content was classified and evaluated based on whether that instruction took place in a rural or urban area (Table 2). Although not statistically significant, within middle school in urban areas, a greater percentage of students were taught sexual education content such as abstinence as a method to prevent pregnancy, abstinence as the most appropriate way to prevent pregnancy, the prevention of STDs, and contraceptive methods except for OCPs and the emergency contraceptive (p>0.05) (Table 2). Despite lacking significance statistically, within high schools in urban areas, a greater percentage of students were taught sexual education content such as abstinence as a method to prevent pregnancy, abstinence as the most appropriate way to prevent pregnancy, male and female anatomy and physiology, and contraceptive methods including condoms, emergency contraceptives, transdermal patches, and permanent methods of sterilization (p>0.05) (Table 2). Within middle and high school, a greater percentage of students in rural areas were taught how to choose which contraceptive method was right for them (p>0.05) (Table 2). A lower percentage of students in rural areas received instruction regarding male and female anatomy and physiology within middle school than in urban areas (p=0.0378) (Table 2).

**Table 2:** Middle and high school sexual education content in rural versus urban areas. This table denotes the sexual education content included in surveyed students’ middle and high school curricula based on the population density of the schools’ locations. The most common topics students were taught in middle school sexual education curriculums were abstinence as a method to prevent pregnancy (50.00%) in rural areas and male and female anatomy and physiology (62.30%) in urban areas. The second most common topic students attending middle school were taught was that abstinence was stressed as the most appropriate way to prevent pregnancy (44.44%) in rural areas and abstinence as a method to prevent pregnancy (61.48%) in urban areas. In terms of contraception content, middle school sexual education programs primarily included information regarding condoms in rural (27.78%) and urban areas (41.80%). The most common topic students were taught in high school sexual education curriculums was prevention of STDs (71.43%) in rural areas and abstinence as a method to prevent pregnancy (67.46%) in urban areas. The second most common topics high school students were taught was abstinence as a method to prevent pregnancy (57.14%), and male and female anatomy and physiology (57.14%) in rural areas, and the prevention of STDs (65.87%) in urban areas. In terms of contraception content, high school sexual education programs primarily included information regarding condoms in rural (50.00%) and urban areas (55.56%). There was a statistical significance in instruction on male and female anatomy and physiology between rural and urban areas (p=0.0378) within middle school. Otherwise, there was no statistical significance (p > 0.05) between rural and urban sexual education content in high school or middle school.

Sexual education content	Percentage of students (%)						
	Middle school			High school			
	Rural	Urban	p-value	Rural	Urban	p-value	
Abstinence as a method to prevent pregnancy	50.00 (N=9)	61.48 (N=75)	0.4412	57.14 (N=8)	67.46 (N=85)	0.5520	
Abstinence was stressed as the most appropriate way to prevent pregnancy	44.44 (N=8)	51.64 (N=63)	0.6205	42.86 (N=6)	50.00 (N=63)	0.7796	
Prevention of sexually transmitted diseases (STDs)	33.33 (N=6)	41.80 (N=51)	0.6108	71.43 (N=10)	65.87 (N=83)	0.7734	
Male and female anatomy and physiology	33.33 (N=6)	62.30 (N=76)	0.0378	57.14 (N=8)	62.70 (N=79)	0.7739	
Information about the usage, safety and/or effectiveness of condoms	27.78 (N=5)	41.80 (N=51)	0.3102	50.00 (N=7)	55.56 (N=70)	0.7804	
Information about the usage, safety and/or effectiveness of oral contraceptives (OCPs) (oral birth control)	22.22 (N=4)	17.21 (N=21)	0.7411	35.71 (N=5)	32.54 (N=41)	0.7734	
Information about the usage, safety and/or effectiveness of emergency contraception (the morning after pill)	11.11 (N=2)	8.20 (N=10)	0.6534	14.29 (N=2)	22.22 (N=28)	0.7341	
Information about the usage, safety and/or effectiveness of spermicide	5.56 (N=1)	9.02 (N=11)	>0.9999	28.57 (N=4)	16.67 (N=21)	0.2768	
Information about the usage, safety and/or effectiveness of hormonal intrauterine devices (IUDs)	5.56 (N=1)	9.84 (N=12)	>0.9999	28.57 (N=4)	19.05 (N=24)	0.4794	
How to choose which contraceptive method was right for you	5.56 (N=1)	3.28 (N=4)	0.5029	14.29 (N=2)	10.32 (N=13)	0.6472	
Information about the usage, safety and/or effectiveness of female condoms	0 (N=0)	12.30 (N=15)	0.2175	28.57 (N=4)	19.84 (N=25)	0.4884	
Information about the usage, safety and/or effectiveness of the contraceptive diaphragm	0 (N=0)	9.84 (N=12)	0.3641	35.71 (N=5)	15.87 (N=20)	0.1319	
Information about the usage, safety and/or effectiveness of the vaginal sponge	0 (N=0)	3.28 (N=4)	>0.9999	28.57 (N=4)	9.52 (N=12)	0.0566	
Information about the usage, safety and/or effectiveness of contraceptive injections (the shot)	0 (N=0)	5.74 (N=7)	0.5949	28.57 (N=4)	15.08 (N=19)	0.2474	
Information about the usage, safety and/or effectiveness of copper intrauterine devices (IUDs)	0 (N=0)	6.56 (N=8)	0.5962	28.57 (N=4)	15.87 (N=20)	0.2611	
Information about the usage, safety and/or effectiveness of subdermal implants	0 (N=0)	3.28 (N=4)	>0.9999	14.29 (N=2)	8.73 (N=11)	0.6201	
Information about the usage, safety and/or effectiveness of transdermal patches	0 (N=0)	1.64 (N=2)	>0.9999	0 (N=0)	7.94 (N=10)	0.5981	
Information about the usage, safety and/or effectiveness of vaginal rings	0 (N=0)	4.10 (N=5)	>0.9999	21.43 (N=3)	10.32 (N=13)	0.2021	
Information about permanent methods of contraception including vasectomies and tubal ligation	0 (N=0)	4.92 (N=6)	>0.9999	14.29 (N=2)	15.08 (N=19)	>0.9999	

**Figure 4:**
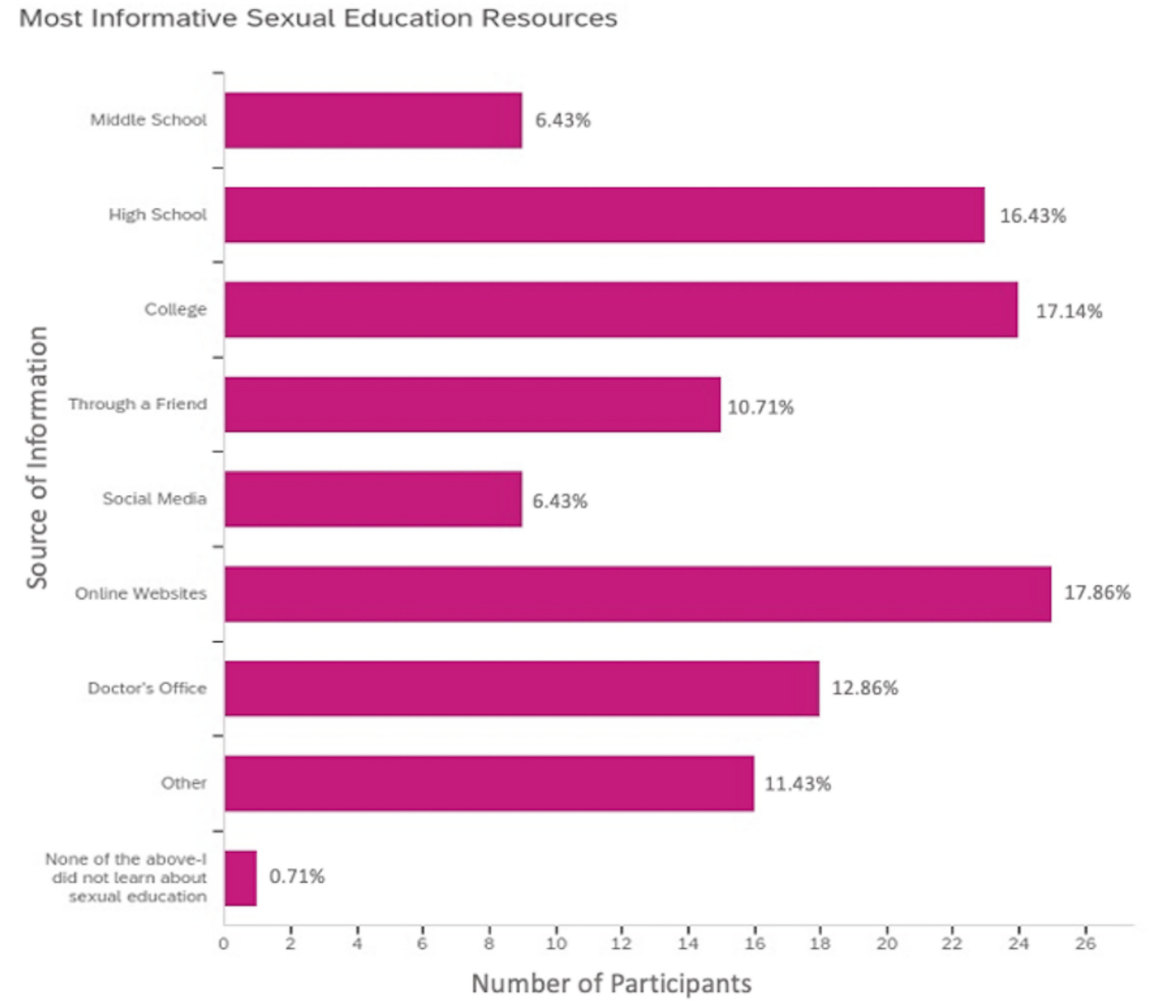
Primary sources of sexual education and contraceptive information. Students received most sexual education information from online websites, within college, or during high school. Fewer students stated that they obtained sexual education information via social media and middle school. Surveyed students who listed, “Other,” indicated that their parents taught them information relating to sexual education or that they learned this information from medical school, sexual partners/friends, a single seminar/class, or within 5th grade.

Surveyed students learned their most informative sexual education information from a variety of sources including formal classroom instruction within middle (6.43%; N=9) and high school (16.43%; N=23), college (17.14%; N=24), through a friend (10.71%; N=15), through social media (6.43%; N=9), through online websites (17.86%; N=25), and at the doctor’s office (12.86%; N=18) (Figure 4). Over 10% of respondents reported they received their most informative sexual education from sources other than those that were listed (11.43%: N=16) (Figure 4). Out of the 16 respondents who reported learning most information from other sources, 37.5% (N=6) indicated that their parents taught them information relating to sexual education or that they learned this information from medical school (18.75%; N=3), sexual partners/friends (12.5%; N=2), a single seminar/class (12.5%; N=2) or within 5th grade (18.75%; N=3) (Figure 4). Less than one percent of students reported they did not learn about sexual education at all (0.71%; N=1) (Figure 4). A greater percentage of students received their most informative sexual education resources within high school over middle school (16.43%; N=23 in high school and 6.43%; N=9 in middle school) (Figure 4).

**Figure 5:**
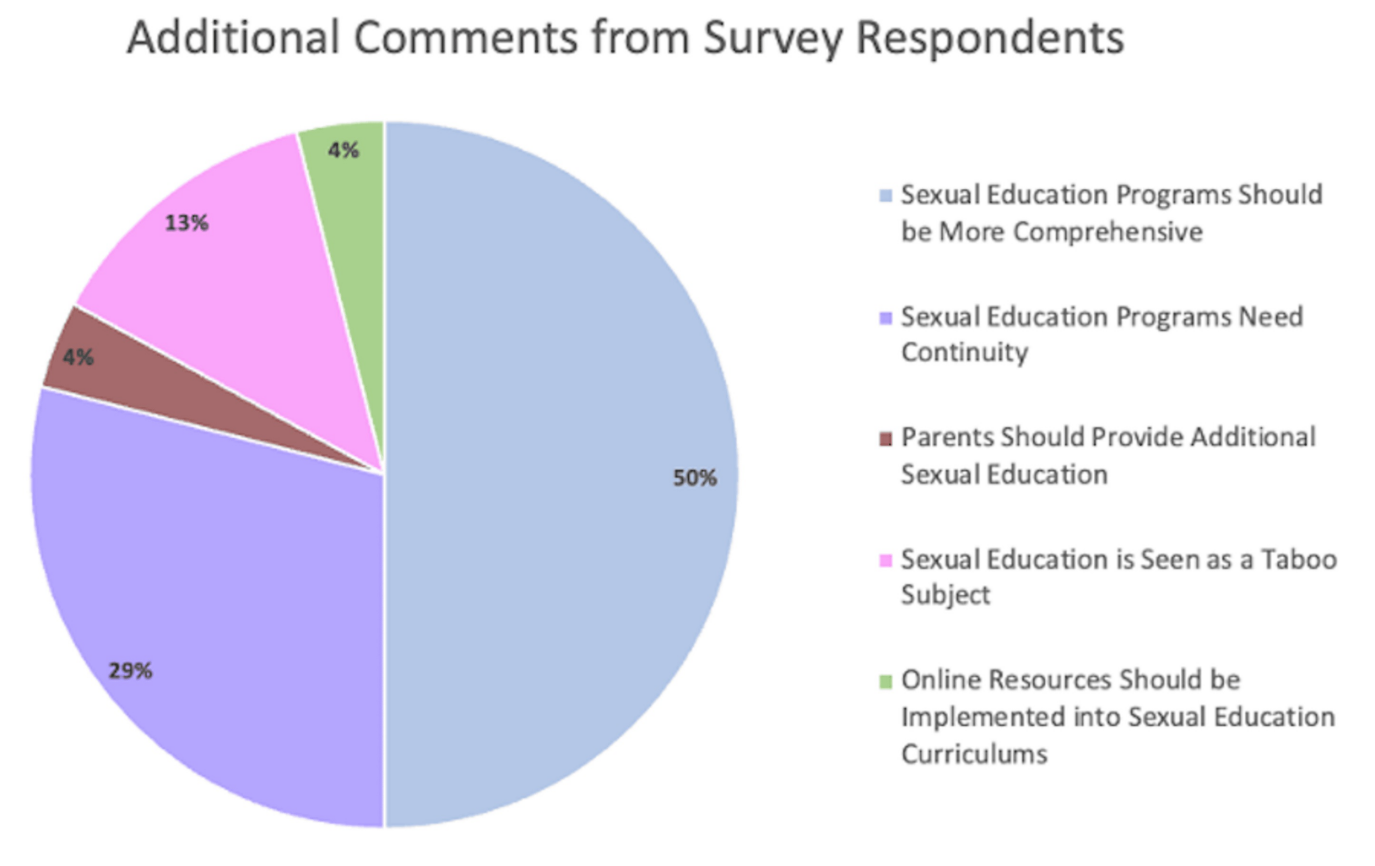
Additional comments of survey respondents provided at the conclusion of the survey. Based on response content, a total of 24 comments were evaluated and separated into one of five categories including sexual education programs should be more comprehensive (50.00%), sexual education programs need continuity (29.17%), parents should provide additional sexual education (4.17%), sexual education is seen as a taboo subject (12.50%), and online resources should be implemented into sexual education curriculums (4.17%).

Any additional comments survey participants had at the conclusion of the survey were evaluated and separated into five separate categories (Figure 5). The most prevalent comment detailed concerns that sexual education programs should be more comprehensive (50.00%; N=12) whereas the least frequent comments stated that parents should provide additional sexual education outside of a formal classroom setting (4%; N=1) and online resources should be incorporated into the current sexual education curriculums within schools (4%; N=1) (Figure 5). The second most popular comment stated sexual education should be provided continuously throughout each grade level of middle and high school curriculums (29.17%; N=7) (Figure 5). Other respondents noted that given the current way sexual education is presented, it is often thought of as a taboo subject (13%; N=3) (Figure 5).

## Discussion

This survey encapsulated data and views from osteopathic medical students with diverse backgrounds who primarily attended public middle and high schools throughout the United States within rural-urban residences (Figure 1). Most participants lived in urban areas in the South during middle and high school. However, the diverse responses suggest that the findings may be applicable to public sexual education curricula nationwide, irrespective of rural-urban residence.

The primary objective of this survey relates to the age of initiation and content of sexual education programs at middle and high schools throughout the United States. The age of initiation of sexual education curriculum has remained largely unchanged from 1988 to 2019 [2]. Most surveyed students received sexual education curriculums within the ninth grade, but some received instruction as early as fifth grade (Figure 2A). As students continued into high school, they progressively received less sexual education instruction (Figure 2A). Very few students received a sexual education curriculum throughout the entire course of middle and high school (Figure 2A) and fewer students reported receiving their most informative sexual education content within middle school as opposed to high school (Figure 4). Similarly to the results in this study, most sexual education is not taught continuously throughout middle and high school curriculums [3]. Students receive the most formal sexual education in high school compared to during middle or elementary school [9]. Since the late 1900s, adolescents have received formal sexual education as early as grade five (10 to 11 years old), but most states have non-specific guidelines stating that sexual education should be initiated at an age-appropriate time with some mentioning that initiation occurs anywhere from grade six to 12 [2]. Within this study, most students reported receiving contraception education specifically within middle school from ages 10 to 14 (Figure 2B), which aligns with the Centers for Disease Control and Prevention (CDC)’s guidance of contraception initiation at age 13-14 [1]. The American College of Obstetricians and Gynecologists (ACOG) recommends that the initial reproductive health visit take place between ages 13 and 15 to discuss contraception and STDs or earlier based on each individual’s needs regardless of their age or whether they’ve been sexually active [10]. However, despite this guidance almost half of students reported that they did not think their sexual education curriculum was taught to them at an appropriate age (Figure 3). These students mentioned that their sexual education was taught too late in the curriculum and stated they would have preferred initiation as early as age 12. These comments, along with additional comments left at the conclusion of the survey (Figure 5), suggest that sexual education should be given continuously throughout middle and high school education with instruction beginning early on within middle school education. Corroboratively, most teenage pregnancies occur in females aged 15 to 19 [11] which correlates to teens in grades 10 through 12. Given the high rates of teenage pregnancy among adolescents aged 15 to 19, earlier introduction of sexual education may be beneficial. When adolescents undergo puberty, they are challenged with newfound fertility and have questions regarding anatomy and sexuality, including gender and sexual identities, which ultimately affect their physical, mental, and emotional well-being [12]. Additionally, during this time they may begin sexual experimentation, and because of poor risk/consequence assessment skills, they may be impacted by unintended pregnancies [12]. Thus, these questions regarding reproductive anatomy and sexuality should be addressed as early as age 10 in a comprehensive sexual education program that includes the aforementioned topics alongside contraception, STD prevention, consent, and interpersonal relationships in efforts to mitigate consequences resulting from late initiation of relevant sexual health information [12,13].

Additionally, although most students reported their sexual education curriculum was given at an appropriate age (Figure 3), they mentioned this education lacked comprehensibility (Figure 5) including relevant information concerning sexual health as it focused on abstinence or exclusively on anatomy without instruction on contraception. Historically, this aligns with national data reporting that many schools either exclusively teach abstinence-only methods with or without contraception education or emphasize abstinence as the preferred method [2]. Similarly, according to survey responses regardless of grade level in school, students who received sexual education content were primarily taught abstinence-only instruction or abstinence as a method and male and female anatomy and physiology (Table 1). Students received less sexual education instruction about STD prevention and information about all contraceptive methods including condoms, OCPs, female condoms, hormonal and copper IUDs, contraceptive diaphragm, spermicide, emergency contraception, contraceptive injections, permanent sterilization methods, vaginal rings, vaginal sponge, subdermal implants, and transdermal patches in middle school (Table 1). Within middle school, fewer students were taught how to choose which contraceptive method was right for them (Table 1). To adequately do this, adolescents should be provided with sufficient supplemental resources involving websites, other educational materials, and/or access to support groups to evaluate the different contraceptive methods themselves outside of a formal classroom setting [14]. Irrespective of secondary school status, students received very little instruction on any of the contraceptive methods except for information about condoms and occasionally OCPs (Table 1). In fact, students reported they received their most informative sexual education content primarily from sources other than secondary instruction such as online websites and colleges (Figure 4). Given that most students reported learning about sexual education primarily from sources other than secondary school instruction, there is a need for reform of current curriculums towards the adoption of a more comprehensive model that integrates online resources such as self-study websites [2]. Based on the National Survey of Family Growth in 2002, one study suggests that those adolescents who received comprehensive sexual education had lower rates of reported teen pregnancies and delayed initiation of vaginal intercourse as compared to abstinence-only education [5]. This study also concluded that teaching about contraception was not associated with an increased risk of adolescent sexual activity or STDs [5]. Given these benefits of comprehensive sexual education, we are currently missing a critical opportunity to properly provide this education to students within their secondary school institutions. Comprehensive sexual education curriculums including both abstinence and contraception help to delay first sexual encounters, decrease the frequency of sexual activity and number of sexual partners, and help teens have healthy relationships and avoid STDs and unintended pregnancies with reduced sexual risk-taking [9]. Those curriculums that solely focus on abstinence or do not teach about contraception methods put youth at increased risk of pregnancy and STDs as they do not stop or delay sexual encounters [9]. Based on data from the National Longitudinal Survey of Youth 1979, if teenagers become pregnant they are at increased risk of poor physical and mental health outcomes later in life regardless of whether the pregnancy results in a live birth [15]. Therefore, if we are aware of the benefits of comprehensive sexual education and implement such curriculums into secondary school instruction, we can act early to try to mitigate the impact an unintended pregnancy may have on women’s mental health.

Moreover, the way in which the curriculum is taught must be addressed. Some students mentioned that the way in which their sexual education was provided made sexual health a taboo or stigmatized subject and made them feel uncomfortable speaking about sexual health (Figure 5). They mentioned that this ultimately made it harder to look for appropriate resources when they eventually chose to have sex. This same concept has been alluded to by others in prior studies [16]. These comments ultimately refer to fear-based sexual education teachings which involve scare tactics, lack of contraception education, medical misinformation, focus on negative consequences of sexual behavior images, religious bias, and omissions in sexual orientation and diversity [17]. In future curricula reform, special attention should be made to ensure greater inclusivity along with language modifications to create safe spaces for adolescents to discuss sexual health.

Creation and maintenance of an appropriate comprehensive and inclusive sexual education at an age-appropriate time can be a difficult and daunting task. Based on these survey results, we can propose several modifications to current sexual education practices. One suggestion would be to initiate a comprehensive sexual education curriculum within early middle school progressively by increasing content exposure in high school education. For example, initial sexual education content should involve a combination of male and female anatomy and physiology with progression to include STDs, abstinence instruction, interpersonal relationships, consent, topics surrounding sexuality, and then contraception information as the students age within middle school with presentation of these topics in greater complexity as they age towards high school via separate yearly courses. This information can be relayed in a formal classroom setting as well as by healthcare professionals utilizing social media and links to self-study websites to engage adolescent interest [2]. As suggested by the comments at the conclusion of this survey (Figure 5), parents may also play a supplementary role in the delivery of sexual education information [18]. Based on a qualitative study of parents’ opinions, most agreed with the notion of teaching comprehensive sexual education, but research has suggested that parents often lack the necessary knowledge to effectively teach this education to their children at home [16]. One attempted intervention suggested parental involvement via the usage of mobile and online technologies in efforts to optimize parental and child engagement in sexual education [19]. Yet, this trial concluded that to optimize results, such intervention should be implemented alongside school and teacher communication with parents regarding sexual education topics [19]. Furthermore, given that most surveyed students reported they learned their most informative sexual education content through the utilization of electronic resources including online websites, future formal secondary sexual education programs should aim to implement such resources into current curriculums. There have been several self-study websites that include information on STDs, physiology, and contraception that aid women in choosing a method based on their preferences [2]. The incorporation of such self-study websites may be instrumental in bolstering students’ ability to learn and retain sexual education information as they progress through secondary schooling and beyond. Medical students may also play a valuable role in educating youth. One study has suggested that medical students can help bridge the gap in sexual education specifically regarding youth within middle schools [20]. This study reported that the middle school students who received instruction from medical students had a better understanding of reproductive system anatomy and community resources as well as improved sexual decision-making [20]. It was noted that more than half of middle school teachers while less than 20% of medical students reported discomfort in teaching about sexual health [20]. Thus, the delivery of sexual education from medical students who are often closer in age to middle and high school students compared with classroom teachers provides youth with a more relaxed and inclusive setting with instructors who are more comfortable teaching about sexual health.

The secondary objective of this survey involves a comparison of sexual education content within middle and high schools based on rural-urban residence. Overall, there was no statistically significant difference in contraception education between rural and urban areas (p<0.05) (Table 2). Consequently, there can be no solid conclusions made suggesting that contraceptive education is lacking for those individuals living in rural vs urban regions. However, within middle schools in urban areas, numerical variation suggests students received more information regarding abstinence instruction, STD prevention, and most contraceptives excluding OCPs and emergency contraceptives (p>0.05) (Table 2). Within high schools in urban areas, numerical variation suggests students received more information regarding abstinence instruction, anatomy, STD prevention, and most contraceptives excluding condoms, emergency contraceptives, transdermal patches, and permanent methods of sterilization (p>0.05) (Table 2). Despite the lack of statistical significance, this represents a potential point of improvement for middle and high school sexual education curricula in rural areas. Numerical variations also suggest that within middle and high school, a greater percentage of students in rural areas were taught how to choose which contraceptive method was right for them (p>0.05) (Table 2). Despite the lack of statistical significance, this represents a potential point of improvement for middle and high school sexual education curricula in urban areas. In contrast, there was a statistically significant greater percentage of students who received instruction on male and female anatomy and physiology within middle schools located in urban areas (p=0.0378) (Table 2). This represents a point of improvement for future rural sexual education curriculums within middle school. Studies have shown that with improved knowledge of anatomy and physiology, and comprehensive sexual education in general, there are lower rates of both STDs and teenage pregnancies [9,21]. Thus, the inclusion of this information is pivotal in improving health outcomes for youth regardless of whether they reside in rural or urban residences.

We acknowledge several shortcomings in this study. First, the sample size and acquired survey responses were limited which could contribute to a lack of statistical significance when evaluating differences between education content within rural-urban residences. Within the surveys, the definition of population density that constituted areas as rural or urban was based on the 2010 Census Bureau definitions. There are updated guidelines released in 2020 that have expanded the definition of urban areas to include populations greater than 5,000 [7]. Utilization of this new definition may have resulted in a more diverse representation of participants when selecting whether they attended middle/high school in rural or urban residences. Another potential limitation exists based on the demographic population of the survey. Survey respondents are medical students, so an extended period of time has passed since participants received formal sexual education within the secondary school which may potentially introduce recall bias. However, when these students were receiving sexual education instruction as adolescents, they were subject to the same curriculum as other adolescents who did not choose a career in medicine. Therefore, the sexual education curricula participants received should not significantly vary from the general population of other adolescents solely because they chose a career in medicine later in life. Ultimately, despite the participants' high level of education and the time gap between their sexual education and this survey, sexual education reform remains a priority, as supported by prior literature on comprehensive sexual education policies [2]. Therefore, even though this is a highly educated subgroup, the results likely reflect the broader population, aligning with previous research findings. To alleviate these shortcomings, future studies should aim to address the above three limitations by expanding on the number of survey participants across the United States within a setting other than medical school such as within large undergraduate institutions given their ability to gather individuals from a diverse rural-urban residence as well as their students’ proximity in time to formal secondary school sexual education instruction.

## Conclusions

This survey encapsulated data and views from 140 osteopathic medical students with diverse backgrounds who attended primarily public middle and high schools throughout the United States within rural-urban residences. Based on survey responses from highly educated students and in conjunction with prior literature reviews, we can assume that comprehensive sexual education curriculum reform is necessary and may be initiated within middle school with continuation into high school. Efforts should be made to incorporate online resources into future formal secondary sexual education programs. Additionally, special attention should be made to ensure greater inclusivity along with language modifications to create safe spaces for adolescents to discuss sexual health.

This survey also seeks to compare sexual education content within middle and high schools based on rural-urban residence. However, there was no statistically significant difference in contraception education between rural and urban areas. Consequently, there can be no solid conclusions made suggesting that contraceptive education is lacking for those individuals living in rural vs urban regions. Numerical variation in results suggests that students within middle and high schools in urban areas received more information regarding abstinence instruction, STD prevention, and some contraceptives. Despite the lack of statistical significance, this represents a potential point of improvement for middle and high school sexual education curricula in rural areas. As it relates to general sexual education, there were more students who received instruction on male and female anatomy and physiology within middle schools located in urban areas. Future studies should aim to expand the number of survey participants across the United States within a setting other than medical school such as within large undergraduate institutions given their ability to gather individuals from a diverse rural-urban residence as well as their students’ proximity in time to formal secondary school sexual education instruction.

## Appendices

Survey

1. Which is your graduating class? Choices- 2026, 2025, 2024, 2023

2. Sex assigned at birth: Choices- Male or female. Other? ____ (allow space to type answer)

3. What is your current age? Choices- 18-24 years old, 25-30 years old, 31-35 years old, 36 years old and up.

4. In which state did you attend middle school? -Include a drop-down menu with all of the states listed.

5. In which state did you attend high school? -Include a drop-down menu with all of the states listed.

6. Did you attend public school or private school? Choices- Public School or Private School.

7. Did you attend middle school in a rural or urban area? Urban area definition: areas with 2,500 people or more. Rural area definition: area with less than 2,500 people (US Census definition). Choices- Rural area or urban area.

8. Did you attend high school in a rural or urban area? Urban area definition: areas with 2,500 people or more. Rural area definition: area with less than 2,500 people (US Census definition). Choices- Rural area or urban area.

9. Did your middle or high school include sexual education in their curriculum? Choices- Yes or No

10. In which grade level(s) was sexual education presented to you in the curriculum at the middle/high school you attended? (Choose all that apply) Choices- 5th grade, 6th grade, 7th grade, 8th grade, 9th grade, 10th grade, 11th grade, 12th grade, none of the above, all of the above.

11. At what age did you learn about different contraception methods? Choices- 10 years old, 11 years old, 12 years old, 13 years old, 14 years old, 15 years old, 16 years old, 17 years old, 18 years old, 19 years old, none of the above-I did not learn about different contraceptive methods.

12. Was the sexual education provided to you as a separate class or given as a unit within another class? Choices- Separate class or unit within another class

13. Which information was included/taught within your middle school’s sexual education curriculum? Select all that apply. (Give drop down menu with all of the following choices.)

(a) Prevention of sexually transmitted diseases (STDs).

(b) Male and female anatomy and physiology.

(c) Abstinence as a method to prevent pregnancy.

(d) Abstinence was stressed as the most appropriate way to prevent pregnancy

(e) Information about the usage, safety and/or effectiveness of condoms.

(f) Information about the usage, safety and/or effectiveness of female condoms.

(g) Information about the usage, safety and/or effectiveness of the contraceptive diaphragm.

(h) Information about the usage, safety and/or effectiveness of spermicide.

(i) Information about the usage, safety and/or effectiveness of the vaginal sponge.

(j) Information about the usage, safety and/or effectiveness of contraceptive injections (the shot).

(k) Information about the usage, safety and/or effectiveness of hormonal intrauterine devices (IUDs).

(l) Information about the usage, safety and/or effectiveness of copper intrauterine devices (IUDs).

(m) Information about the usage, safety and/or effectiveness of subdermal implants.

(n) Information about the usage, safety and/or effectiveness of oral contraceptives (OCPs) (oral birth control).

(o) Information about the usage, safety and/or effectiveness of emergency contraception (the morning after pill).

(p) Information about the usage, safety and/or effectiveness of transdermal patches.

(q) Information about the usage, safety and/or effectiveness of vaginal rings.

(r) Information about permanent methods of contraception including vasectomies and tubal ligation.

(s) How to choose which contraceptive method was right for you.

14. Which information was included/taught within your high school’s sexual education curriculum? Select all that apply. (Give drop down menu with all of the following choices.)

(a) Prevention of sexually transmitted diseases (STDs).

(b) Male and female anatomy and physiology.

(c) Abstinence as a method to prevent pregnancy.

(d) Abstinence was stressed as the most appropriate way to prevent pregnancy

(e) Information about the usage, safety and/or effectiveness of condoms.

(f) Information about the usage, safety and/or effectiveness of female condoms.

(g) Information about the usage, safety and/or effectiveness of the contraceptive diaphragm.

(h) Information about the usage, safety and/or effectiveness of spermicide.

(i) Information about the usage, safety and/or effectiveness of the vaginal sponge.

(j) Information about the usage, safety and/or effectiveness of contraceptive injections (the shot).

(k) Information about the usage, safety and/or effectiveness of hormonal intrauterine devices (IUDs).

(l) Information about the usage, safety and/or effectiveness of copper intrauterine devices (IUDs).

(m) Information about the usage, safety and/or effectiveness of subdermal implants.

(n) Information about the usage, safety and/or effectiveness of oral contraceptives (OCPs) (oral birth control).

(o) Information about the usage, safety and/or effectiveness of emergency contraception (the morning after pill).

(p) Information about the usage, safety and/or effectiveness of transdermal patches.

(q) Information about the usage, safety and/or effectiveness of vaginal rings.

(r) Information about permanent methods of contraception including vasectomies and tubal ligation.

(s) How to choose which contraceptive method was right for you.

15. Where did you receive most information about topics related to sexual education? Ex: Contraception. Choices- Middle School, High School, College, Through a Friend, Social Media, Online Websites, Doctor’s Office, Other____ (allow space to type answer), None of the above-I did not learn about sexual education.

16. Do you think the sexual education at your middle and/or high school was presented to you at an early enough age? Choices- Yes or No.

(a) If participants answered "No" to previous question (#16): When do you think you should have been exposed to sexual education/birth control education? Choices- 10 years old, 11 years old, 12 years old, 13 years old, 14 years old, 15 years old, 16 years old, 17 years old, 18 years old, 19 years old (or can give grade level).

17. Additional Comments: _______. (allow space to type answer)
